# The mitochondrial genome of the sooty swift (*Cypseloides fumigatus*)

**DOI:** 10.1080/23802359.2017.1307702

**Published:** 2017-04-05

**Authors:** Renata Neves Biancalana, Cibele Biondo, Fabio Raposo do Amaral

**Affiliations:** a Centro de Ciências Naturais e Humanas, Programa de Pós-graduação em Evolução e Diversidade, Universidade Federal do ABC, Alameda da Universidade, São Bernardo do Campo, São Paulo, Brazil;; b Departamento de Ecologia e Biologia Evolutiva, Universidade Federal de São Paulo, Diadema, São Paulo, Brazil

**Keywords:** Cypseloidinae swifts, Neotropics, ultraconserved elements

## Abstract

We assembled the mitogenome of *Cypseloides fumigatus* based on off-target sequences from ultraconserved elements sequencing. We found a total length of 16,850 bp, including 13 protein-coding genes, 22 tRNA genes, 2 rRNA genes, and one control region, organized in the standard avian gene order. We have built a phylogenetic tree including 26 species of swifts that suggested *C. fumigatus* as sister species of *C. cryptus,* and indicated exciting opportunities for biogeographic inferences involving most continents, including Neartic vs Neotropical disjunctions and local radiations across the globe. Finally, we found cases of lack of reciprocal monophyly between named species and high intra-specific divergence, suggesting that population-level studies are warranted.

Swifts and swiftlets (family Apodidae) comprise a globally distributed group of small to medium-sized insectivorous birds closely related to hummingbirds. Examples of interesting aspects of their biology include their high-speed flight, large portion of their lifetime spent in flight and little variation in plumage colouration (Chantler & Driessens 2000). Here we present the complete mitogenome of the Sooty Swift (*Cypseloides fumigatus*), a relatively rare species of the Cypseloidinae subfamily distributed in Argentina, Bolivia, Brazil, and Paraguay. This is the third swift genome sequenced to date, the other being *Apus apus* (Morgan-Richards et al. 2008, NC_008540.1) and *Chaetura pelagica* (Xu & Zhang 2015, NC_028545.1). We obtained genomic DNA from a muscle sample of an individual collected at Ortigueira, PR, Brazil (24°12′S 50°55′ W, deposited at LGEMA USP tissue collection under #11411), using the Qiagen DNeasy kit (Valencia, CA) with an RNAse treatment. We obtained the mitogenome (Genbank KY688216) from off-target sequences generated during next-generation sequencing of ultraconserved elements (UCEs, performed at Rapid Genomics LLC, Gainesvile, FL), as described in Amaral et al. (2015). We manually aligned four fragments resulting from the mtDNA mining protocol, whose lengths varied from 14,923 bp to 16,872 bp., in Bioedit (Hall 1999). We obtained a final consensus sequence of 33,722 bp, which we used as input for automatic annotation in DOGMA (Wyman et al. 2004; http://dogma.ccbb.utexas.edu) and MITOS (Bernt et al. 2013; http://mitos.bioinf.uni-leipzig.de/index.py) using default parameters. We manually adjusted the automatic annotations using the mitogenome of *Apus apus* as reference to resolve discordances between the two automatic methods.

We obtained an annotated mitogenome of 16,850 bp, 187 bp shorter than the genome of *Apus apus*, and that included 2 rRNAs, 22 tRNAs, 13 protein-coding genes, and 1 control region. Gene order was the same found in *Apus apus,* which is identical to one thought to be ancestral among birds (Gibb et al. 2007). Base composition (A = 31.0%, C = 30.6%, G = 13.6%, and T = 24.8%) was similar to that reported for *Apus apus*. Interestingly, NADH dehydrogenase subunit 3 in *C. fumigatus* had an extra untranslated base, which has been reported in a number of bird species (Mindell et al. 1998) but is absent in the two other published swift mitogenomes. The control region has a long stretch of repetitive sequences precluded establishing the exact number of repetitions, and thus the exact length of the mitogenome may vary slightly from one reported here.

We perfomed a phylogenetic analysis including 622 bp of COI from the sequence obtained here and other 25 Apodidae species available at Genbank, totalling 83 sequences. We aligned the sequences using ClustalW (Larkin et al. 2007) as implemented in Geneious 9.1.6 (Kearse et al. 2012). We carried out model selection using Akaike information criterion in MrModeltest 2 (Nylander 2004). We performed a Bayesian phylogenetic inference in MrBayes 3.2.6 (Huelsenbeck & Ronquist 2001) using GTR + I + G, 10.000.000 generations, sampling frequency of 5000, burnin of 25% and *Lophornis magnificus* as an outgroup. The phylogenetic analysis indicated that *C*. *fumigatus* and *C*. *cryptus* are sister species (Figure 1), suggesting a split between eastern and western Neotropics. Despite limited resolution, our tree also suggested interesting historical events as Neotropical vs Neartic disjunctions (e.g. clade *Tachornis*/*Aeronautes*), local radiations entirely or mostly restricted to the Old World (e.g. *Apus*), Neotropics (*Chaetura*) and Asia, Oceania and Australia (*Aerodramus*). In addition, we found high intra-specific divergences in *S. zonaris*, *C. cinereiventris* and haplotype sharing among *Apus* species, what indicate exciting opportunities for population-level studies.

**Figure 1.**
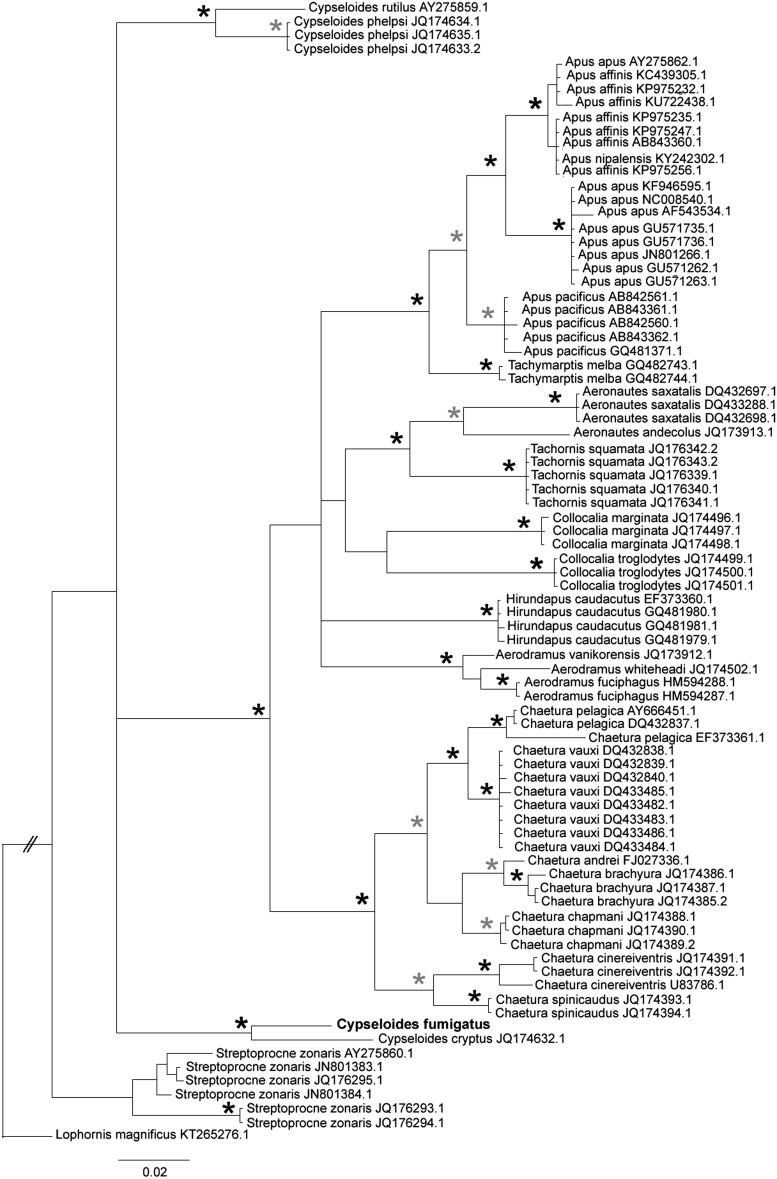
Bayesian phylogenetic inference based on COI sequences of swifts. Accession numbers are indicated at the right of species names. Stars correspond to posterior probabilities of 1.0 (black), or equal to or within the interval of 0.95 to 0.99 (gray). Posterior probabilities lower than 0.95 were not indicated.
